# Delivering multi-disease screening to migrants for latent TB and blood-borne viruses in an emergency department setting: A feasibility study

**DOI:** 10.1016/j.tmaid.2020.101611

**Published:** 2020

**Authors:** Sally Hargreaves, Laura B. Nellums, Catherine Johnson, Jacob Goldberg, Panagiotis Pantelidis, Asif Rahman, Jon S. Friedland FMedSci

**Affiliations:** a Institute for Infection & Immunity, St. George's, University of London, United Kingdom; b Imperial College Healthcare NHS Trust, London, United Kingdom; c Section of Infectious Diseases & Immunity, Imperial College London, United Kingdom; d Division of Epidemiology and Public Health, School of Medicine, University of Nottingham, Nottingham, United Kingdom

**Keywords:** Latent TB, LTBI, HIV, Hepatitis, Migrant, Europe, Multi-disease screening, Health-service delivery

## Abstract

**Background:**

Screening for latent tuberculosis infection (LTBI) in migrants is important for elimination of tuberculosis in low-incidence countries, alongside the need to detect blood-borne infections to align with new guidelines on migrant screening for multiple infections in European countries. However, feasibility needs to be better understood.

**Methods:**

We did a feasibility study to test an innovative screening model offering combined testing for LTBI (QuantiFERON), HIV, hepatitis B/C in a UK emergency department, with two year follow-up.

**Results:**

96 economic migrants, asylum seekers and refugees from 43 countries were screened (46 [47.9%] women; mean age 35.2 years [SD 11.7; range 18–73]; mean time in the UK 4.8 years [SD 3.2; range 0–10]). 14 migrants (14.6%) tested positive for LTBI alongside HIV [1], hepatitis B [2], and hepatitis C [1] Of migrants with LTBI, 5 (35.7%) were successfully engaged in treatment. 74 (77.1%) migrants reported no previous screening since migrating to the UK.

**Conclusion:**

Multi-disease screening in this setting is feasible and merits being further tested in larger-scale studies. However, greater emphasis must be placed on ensuring successful treatment outcomes. We identified major gaps in current screening provision; most migrants had been offered no prior screening despite several years since migration, which holds relevance to policy and practice in the UK and other European countries.

## 1 Introduction

Migrants (defined as foreign born) in Europe face a disproportionate burden of TB, as well as other priority infectious diseases such as HIV, and hepatitis B and C, compared to host populations, comprising over 70% of cases in some low-incidence countries [[1], [2], [3], [4], [5], [6], [7], [8]]. Amid growing levels of migration to the region over the past two decades [9], renewed focus has been placed on developing and strengthening migrant health screening programmes and improving health-service delivery on arrival. Migrant screening programmes in European countries to date have predominantly focused on single diseases – mainly active TB – [10], yet data suggest that combining screening for multiple key infections at one appointment in migrant groups could be effective, with increased acceptability, uptake, and better treatment outcomes in more integrated approaches [[11], [12], [13], [14], [15]]. Recently published guidelines from the European Centre for Disease Prevention and Control (ECDC) [16] has called for consideration to be given to screening and vaccination for multiple infections in newly arrived migrants to the European Union (EU)/European Economic Area (EEA) on arrival, with a focus on latent TB infection (LTBI), active TB, hepatitis B and C, HIV, vaccine-preventable diseases, and parasitic infections;however the extent to which the recommendations made can be implemented in practice in migrant-receiving EU/EEA countries is as yet unclear.

The majority of TB cases in migrants to low TB-incidence countries in Europe are due to reactivation of LTBI [1,2,17], with the highest rates of reactivation occurring 2–5 years after arrival [18]. Renewed focus has recently been given to delivering targeted LTBI screening and treatment in high-risk groups – including migrant populations – as an effective and cost-effective approach to averting cases of active TB [2,[19], [20], [21]], aligning with WHO's END TB strategy targets [22,23] and new ECDC guidelines(16). Most current guidelines recommend testing for LTBI in new migrants from high-incidence countries (⩾150 per 100,000), with screening and treatment being implemented across Europe in migrant and other high-risk populations [24].

Data suggest high acceptability of screening for LTBI and other infections among migrants [21]. However, formal on-arrival screening programmes miss many migrant groups, with evidence of significant barriers to migrant engagement with primary care where screening is routinely delivered [12,25], and lack of consistency in provision of screening [[26], [27], [28], [29], [30]]. There remain significant evidence-gaps around effective and cost-effective approaches to implementing TB and other infectious diseases screening and ensuring successful treatment outcomes [6,21,24,[31], [32], [33]]. As a result, there is a lack of consensus on optimal approaches to improving the detection and treatment of key infections in migrants across Europe [34].[35].

New and innovative approaches to engaging migrants and delivering screening need to be developed and evaluated. Recent studies have examined the feasibility of implementing LTBI screening in high migrant community settings such as language classes, to increase uptake and treatment [36], which successfully engaged migrants (75% uptake, 85% treatment completion) in 71 LTBI-positive students (n = 440). Hospital emergency departments, where migrants are known to be over-represented due to barriers to registering with primary-care providers in several EU/EEA countries, potentially present a good opportunity to deliver cost-effective, accessible screening to migrants. Although routine testing for HIV is encouraged in this setting [37], in practice this is ad hoc in the UK and elsewhere. Additionally, there is no evidence available as to the feasibility of offering LTBI and/or multi-disease testing in emergency departments, although research in this area is currently underway in primary care [38].

We therefore did a feasibility study to investigate the delivery of an innovative opportunistic screening model offering new migrants multi-disease screening using a one-stop blood test for LTBI combined with HIV and hepatitis B/C in an emergency department setting. We also determined the impact of any previous screening for infection.

## 2 Materials and methods

### 2.1 Screening intervention and setting

We developed an innovative combined screening model to facilitate the delivery of LTBI screening alongside HIV and hepatitis B/C, via a one-stop blood test offered new migrants (in the UK for <10 years) presenting to the emergency department. The PROMOTE study (PROmoting Migrant One-stop Testing in Emergency departments) was conducted at St. Mary's Hospital Emergency Department, London, UK, which is a high-migrant area representative of many of the Boroughs across London, and where 49.8% of the resident population was born abroad according to the most recently available 2011 UK national population census data [39]. Migrant patients meeting the study inclusion criteria were offered combined infection screening in addition to the standard care they received, and followed-up according to routine care pathways. The screening intervention was a single venesection to test for: (i) LTBI using interferon gamma release assay (QuantiFERON-TB Gold in-tube); (ii) HIV (HIV screening assay); and (iii) Hepatitis B surface antigen test (HBsAg) and (iv) hepatitis C antibody test (anti-HCVAb). Awareness-raising educational sessions and an information leaflet regarding the study were delivered to clinical staff prior to implementing the intervention. Laboratory testing, recording and communicating of results, and referral to follow-up care in specialist services followed routine care pathways. Patient records of participants found to be positive for LTBI or other infections followed up at two years.

The proposed sample size was calculated in relation to the estimated proportion of participants who would screen positive for latent TB. This was informed by our preceding feasibility study implementing the same screening programme in a primary care setting, in which 18.8% of migrants screened positive for latent TB, the most common infection detected [12]. Assuming a 5% significance level and 10% margin of error, we calculated that a minimum sample size of 59 was required to show an estimated LTBI prevalence of 18.8% among migrant participants.

### 2.2 Inclusion criteria and main outcomes

This study included foreign-born individuals aged 18 years or older who had lived in the UK for 10 years or less, prior to which they lived for one year or more outside Western Europe, North America, Australia, and New Zealand. Main outcomes were: presence of an infection, treatment outcome, primary-care registration, and previous screening.

### 2.3 Migrant recruitment

Patients were invited to participate in the research by a research nurse (JG or CJ), who provided information about the research to all patients presenting to the emergency department Monday-Friday between 8am and 3pm, in line with the cut off time during the study period for the laboratory to receive samples. Patients were engaged in the walk-in section of the emergency department, with the research nurse present in the waiting room during the study hours. Acutely ill patients admitted via ambulance were excluded from the study. Patients who were in the emergency department waiting room prior to clinical assessment were provided with an information leaflet, detailed patient information recruitment letter, and consent form, which were available in the dominant languages seen at this emergency department (including Somali, Polish, Gujarati, Arabic, Farsi, and English). Further interpreting services were available if needed through the NHS telephone interpreting service. It was not known which patients in the waiting room were migrants due to lack of routine data collection at NHS services on migrant status, and so denominator data are not available on the total number of migrants who attended the emergency department during the study period. All participants provided written informed consent, and capacity to consent was assessed in line with the UK Mental Capacity Act Framework [40,41].

### 2.4 Screening procedure and follow-up

Participants completed a questionnaire (piloted in this setting) with questions pertaining to time in the UK, nationality, registration with a local primary-care provider, and whether patients had previously been offered any kind of screening since their arrival. They then provided a peripheral venous blood sample, which was obtained by the research nurse. Blood samples were tested by the local NHS laboratories, as per routine practice.

All participants were contacted by the research nurse with their test results within seven days. Patients with positive results were then offered care through standard hospital pathways for the relevant infections. Patient records for participants found to be positive for LTBI or other infections were followed up at 2 years.

### 2.5 Data management and analysis

Participant data were collected and anonymised by the research nurse for analysis, and downloaded into a password protected Excel spreadsheet. Anonymised data were also extracted for the patient population at the emergency department to provide comparison population data. Data analysis was conducted using Stata 14 statistical analysis software [42].

Descriptive analyses were carried out to examine the demographic characteristics of the migrant participants and the other patients presenting to the emergency department during this time period, and to summarise patient screening and treatment outcomes. Bivariate analyses were carried out to examine associations between demographic characteristics, primary-care registration, screening history, and LTBI.

### Ethical approval

Ethical approval was received from the NHS Health Research Authority London – City & East Research Ethics Committee.

## 3 Results

### 3.1 Emergency department patient population

72,279 patients presented to the emergency department during the nine-month recruitment period required to achieve the target sample size (Table 1). 61.3% of patients (44,040) were reported to be from an ethnic minority background (no data on migrant status available at UK emergency departments), including Asian (6,872; 9.5%), Black African or Black Caribbean (8,797; 12.2%), or ‘other’ (28,371; 39.3%). 49.6% (35,825) of patients were female, and 78.7% (56,874) were registered with a GP.

**Table 1:** St. Mary's emergency department attenders during the study period.

Characteristic	n	%
Ethnicity		
White	28,239	39.1
Asian	6,872	9.5
Black African or Black Caribbean	8,797	12.2
Other	28,371	39.3
Gender		
Male	36,454	50.4
Female	35,825	49.6
GP registration		
Yes	56,874	78.7
No	15,405	21.3

### 3.2 Screening participants

96 migrants from 43 different countries were screened (Table 2, Table 3), including economic migrants (migrating for work or study) (n = 57), asylum seekers/refugees (fleeing conflict or persecution and seeking asylum in the UK) (n = 13), family reunion migrants (joining family living in the UK) (n = 20), undocumented migrants (without necessary authorisation or documents) (n = 4), and those who migrated for medical reasons (n = 2). The mean number of years in the UK was 4.8 years (SD 3.2), ranging from 0 to 10 years, in line with the inclusion criteria. 46 participants (47.9%) were women. The mean age was 35.2 years (SD 11.7). 54 participants (76.2%) were of non-white ethnicity. 53 (55.2%) reported being married, 45 (45.8%) had university level education, and 59 (61.5%) were employed. 60 (63.8%) participants had a household income of less than £1592 per month.

**Table 2:** Migration characteristics.

Migration characteristics	n	% (Mean [SD])
Reason for migration		
Economic	57	59.4
Asylum	13	13.5
Family reunion	20	20.8
Undocumented	4	4.2
Medical reasons	2	2.1
Years in UK	96	(4.8 [3.2])
Region of origin		
Eastern Europe/Former Soviet Union	28	29.2
Central/Southern Europe	4	4.2
Middle East	19	19.8
Africa	24	25.0
South America	10	10.4
Asia	11	11.8
Countries of Origin (number of migrants screened)		

Afghanistan [1], Albania [1], Algeria [3], Argentina [1], Bangladesh [1], Bolivia [1], Brazil [7], Brunei [1], Bulgaria [2], China [2], Egypt [3], Eritrea [2], Estonia [2], Ethiopia [1], Ghana [1], Greece [2], Guinea Bissau [1], Hungary [2], India [2], Iran [4], Iraq [6], Kazakhstan [1], Kosovo [1], Latvia [2], Lithuania [1], Malaysia [1], Mauritius [1], Morocco [2], Peru [1], Philippines [2], Palestine [1], Poland [10], Kuwait [3], Romania [7], Russia [2], Samoa [1], Somalia [3], South Africa [1], Sri Lanka [1], Syria [5], Tanzania [1], The Gambia [1], Tunisia [2].

**Table 3:** Socio-demographic and socio-economic characteristics of presenting migrants.

Characteristics	n	% (mean, SD)
**Socio-demographic characteristics**		

Gender		
Male	50	52.1
Female	46	47.9
Age	96	(35.2, 11.7)
Relationship status		
Married	53	55.2
Single	39	40.6
Widowed	1	1.0
Divorced/separated	3	3.1
Ethnicity		
White	42	23.8
Black African or Black Caribbean	9	9.4
Asian	18	18.8
Arab	27	28.1
**Socio-economic characteristics**		
Education		
Below secondary	15	15.6
Secondary	38	38.5
University	45	45.8
Employment		
Employed	59	61.5
Student	9	9.4
Unemployed/Economically inactive	25	26.0
Home	3	3.1
Household income^a^		
£0-420	21	22.3
£421-928	14	14.9
£929-1592	25	26.6
£1593-2471	14	14.9
£2471+	20	21.3

a Gross monthly household income based on 2011 Census. For comparison, current UK average monthly income in 2019 is £2921.50.

### 3.3 Screening results

**Fig. 1:**
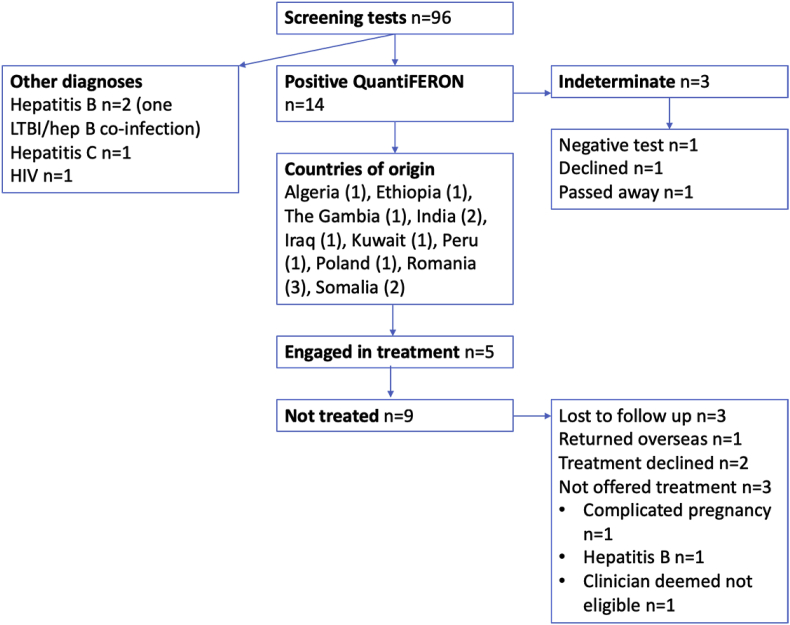
Screening pathways for recruited migrant patients (n = 96).

Screening pathways of migrants recruited to the study are summarised in Fig. 1.

14 migrants (14.6%) had a positive QuantiFERON test (Table 4). Three participants (3.1%) had an indeterminate QuantiFERON result. None of the participants had active TB. There were 2 cases of hepatitis B (1 case of hepatitis B/LTBI co-infection) and 1 case each of hepatitis C and HIV, all of which were previously known.

**Table 4:** Characteristics of migrants testing positive for LTBI.

Age	Gender	Country of origin	Years in UK	GP?	Previously tested	Ethnicity	Household income	Employment status	Education Level	Reason for migration
40	Male	Somalia	5	Yes	Yes	Black African	£421–928 pm	Employed	University	Economic
45	Male	Romania	1	No	No	White	£929–1592 pm	Employed	Below secondary	Economic
36	Male	Algeria	8	Yes	No	Other	£0–420 pm	Employed	University	Economic
53	Female	Poland	9	Yes	No	White	£929–1592 pm	Employed	Secondary	Economic
28	Male	Kuwait	<1	No	No	Other	£1593–2471 pm	Employed	Secondary	Medical treatment
37	Female	Iraq	9	Yes	No	Other	£421–928 pm	Employed	University	Family
52	Male	The Gambia	5	Yes	Yes	Black African	1	Unemployed	Secondary	Economic
34	Female	Romania	3	Yes	No	White	£929–1592 pm	Employed	University	Economic
26	Male	Peru	2	Yes	Yes	Other	£929–1592 pm	Employed	Secondary	Family
38	Female	Romania	2	Yes	Yes	White	£1593–2471 pm	Employed	University	Economic
50	Female	India	6	Yes	Yes	Asian	£2471 + pm	Employed	University	Economic
43	Male	Ethiopia	2	Yes	Yes	Black Caribbean	£421–928 pm	Employed	University	Economic
30	Female	Somalia	10	Yes	Yes	White	£929–1592 pm	At home	Secondary	Asylum
39	Female	India	5	Yes	No	Asian	£1593–2471 pm	At home	Secondary	Family

Five (35.7%) of 14 migrants with a positive QuantiFERON test were subsequently followed-up by specialist services as per routine health-care pathways and were engaged in LTBI treatment, of whom four have successfully completed treatment, and the fifth is currently completing treatment following a pregnancy. Of the other nine participants with positive QuantiFERON tests (64.3%), three (21.4%) were lost to follow-up as they did not attend appointments or could not be contacted. One patient returned overseas, and two declined treatment after follow-up consultations. Three participants were not offered treatment. One of these participants had a complicated pregnancy so LTBI treatment was deferred, and one had a concurrent hepatitis B diagnosis. The third patient was inappropriately discharged from the TB service and not offered latent TB treatment because a clinician deemed them not eligible according to review of the medical records.

Of the three participants (3.1%) with an indeterminate QuantiFERON result, one declined re-testing as she had been previously treated for active TB (the likely explanation for the result), one had a negative screen when retested, and one died from a known unrelated malignancy (this patient had no evidence of active TB).

One patient who screened positive for latent TB was also identified to have hepatitis B. In addition, one further patient screened positive for hepatitis B, one had hepatitis C, and one had HIV, all of which were previously known.

### 3.4 Primary care registration and screening history prior to presentation to the emergency department

20 participants (20.8%) were not permanently registered with a primary-care doctor in the UK, but the majority were. 74 (77.1%) of participants had never been screened for TB or any other infectious disease since migrating to the UK, though recall bias is possible. 19 (95.0%) of these 20 participants not registered with a primary-care provider had never received any infectious diseases screening in the UK (Table 5). Even among the 76 migrants who were registered with a GP, 55 (72.4%) had never been previously screened for TB or any other infectious disease since arrival in the UK. Among participants who screened positive for LTBI [14], 9 (64.3%) had never been previously screened for an infectious disease in the UK.

**Table 5:** Screening history.

	Previous screening in UK	
	Never tested	Previously tested
GP registration		
Not registered	19 (95.0%)	1 (5.0%)
Registered	55 (72.4%)	21 (27.6%)
Test results		
Negative	61 (80.3%)	15 (20.0%)
Positive QuantiFERON	9 (64.3%)	5 (35.7%)
Hepatitis B	1 (50.0%)^a^	1 (50.0%)
Hepatitis C	0 (0.0%)	1 (100.0%)
HIV	0 (0.0%)	1 (100.0%)
Indeterminate	3 (100.0%)	0 (0.0%)

a Positive QuantiFERON.

### 3.5 Correlates of infection, primary care registration, and screening history

We found no association between socio-demographic variables and patients testing positive for LTBI. However, registration with a GP was positively associated with years in the UK (OR: 1.7; 95% CI: 1.3–2.2), with the mean time in the UK for those with a GP being 5.6 years (SD 2.9) compared to 2.0 years (SD 2.6) for those without a GP. Women were more likely to be registered than men (OR 3.2; 95% CI: 1.1–9.3). Registration was also associated with education, with those with secondary or university education being significantly more likely to have a GP (OR: 6.6; 95%: 2.0–21.5). Being previously offered screening was significantly associated with longer time in the UK (1.3; 95% CI: 1.1–1.5), with those tested having been in the UK a mean of 6.6 years (SD 2.8) compared to 4.3 years (SD3.2) among those who had not been screened.

## 4 Discussion

A diverse migrant population presented to this emergency room during the study period, and the multi-disease screening model increased the detection of LTBI in migrants presenting to the emergency department (14 [14.6%]). No cases of active TB were identified, and there was one HIV, two hepatitis B, and one hepatitis C cases identified, though all were previously known. Of the 14 migrants with LTBI, 5 (35.7%) were successfully engaged in treatment within 2 years of follow up although for one patient this was delayed for clinical reasons. 9 (64.2%) did not ultimately receive treatment for a variety of reasons. Only three (21.4%) of participants were lost to follow up. Additionally, two declined treatment after follow-up consultations, one returned to their country of origin, and three were not offered treatment, one of which was due to inaccurate understandings of their eligibility. Importantly, the majority (77.1%) of migrants reported not having been previously offered screening for an infectious disease since migrating to the UK, despite relatively high levels of registration with primary-care providers (79.2%) and being in the UK for an average of 4.8 years. This suggests major gaps in current screening provision to new migrants, and for a need to promote screening for a more diverse range of key infections in the UK.

Our data adds to the growing body of evidence indicating that it is feasible to engage migrants in multi-disease screening including for LTBI. There is mounting evidence of the effectiveness, cost-effectiveness, high uptake, and acceptability of LTBI screening, and combined infectious diseases screening in migrants [12,15,[43], [44], [45], [46], [47]]. In a previous systematic review that we conducted, involving a pooled analysis of migrant screening data from EU/EEA countries, LTBI latent tuberculosis had the highest prevalence across all infections with a median of 15·02% [0·35–31·81]) migrants screening positive for LTBI, a prevalence rate which aligns with this study. The site at which screening is offered may be key, and our data indicate that the emergency department should be considered as a potential potential site for detection, alongside primary care [12,38]. In the UK, Public Health England has advocated for combined packages of infectious disease screening and vaccination for new migrants, aligning with the new ECDC guidelines [16, 48]. The recent ECDC guidelines [16] call for targeted screening for active TB, LTBI, hepatitis B and C, HIV, schistosomiasis, and strongyloidiasis, and to consider the vaccination of newly arrived adult and child migrants to EU/EEA countries for a range of key vaccine-preventable diseases, with ongoing research exploring the feasibility of embedding more integrated screening models into routine primary care and other novel settings. Catch-up vaccination should be offered to adult, adolescent, and child migrants with no evidence of previous vaccinations with MMR (measles-mumps-rubella) and DTP (diphtheria-tetanus-pertussis) vaccines, and to offer hepatitis B vaccination series to all migrant children and adolescents from intermediate (≥2%) or high (≥5%) HBsAg prevalence countries who do not have evidence of vaccination or immunity. Across Europe, migrant communities are facing increasingly restrictive access to health services including primary-care services [49]. This means that in many cases an emergency room remains a migrant's only accessible source of healthcare [50], and are many cases over-represented in such services [51]. Even in this study, with relatively high rates of GP registration, these individuals were accessing emergency services, and had not been previously screened. There is evidence that migrants attending emergency departments may do so for primary care reasons due to the barriers they face within primary care [52,53], however, we did not explore reasons for attendance in our study. We found it difficult to identify migrants in the emergency room context, largely because the NHS on the whole, including emergency departments, does not routinely collect data on migrant status. We relied on individuals in the waiting area to respond to information in leaflets, and identify themselves to the research nurse.

Only three patients were lost to follow up, which was low compared to our previous study engaging migrants for multi-disease screening in primary care [12]. Although previous research has shown high uptake of screening (80%) [21], rates of treatment completion in this and other studies in this area are relatively low [21,27]. LTBI in particular has low rates of treatment completion in comparison to other infections in pooled data from migrant screening programmes across the EU/EEA, with only 54.45% (median, range 35.71–72.27) of migrants diagnosed positive for LTBI ultimately completing treatment after screening [21,27]. Screening programmes will only be effective if we ensure follow-up and linkage-to-care, with migrants are supported along the entire screening and treatment trajectory to minimise drop out.

Our findings show that many migrants accessing emergency services may be missing out on screening for infectious diseases, aligning with previous research and discourse from other EU/EEA countries, despite agreement that it is beneficial [10,38,54,55]. Routine testing for new entrants from high-incidence countries is recommended for LTBI, HIV, and hepatitis B and C in a range of clinical and community settings [22,23,[56], [57], [58]]. Even among those registered with a GP, very few participants in our study reported ever having been offered screening in the UK. This is despite the availability of national guidelines for the screening of high-risk groups – including migrants – in the primary care context and secondary-care settings, particularly for HIV testing, which is being advocated for across healthcare settings including emergency departments [22,59]. This is supported by the work of others: in one study exploring hepatitis B screening in a primary care setting, only 9627 (12%) of 82561 migrants eligible for screening in accordance with national guidelines migrants were offered screening by clinicians, with lack of knowledge and lack of resourced cited by clinicians as key barriers [26].

### 4.1 Strengths and limitations

Whilst this was a UK study, the emergency department model exists across other EU/EEA countries, providing access to walk-in care, often free of charge to migrants who might otherwise be excluded or face barriers to accessing a range of other health service, including primary-care. The presentation of diverse migrant groups in these settings and the infrastructure are thus representative of systems currently in place in Europe, and the findings are likely to be generalisable to emergency departments in other high-migrant receiving countries, which in several European countries now represent a migrant's only source of health care. We are aware that the number of participants in this study is small and that selection bias could have been an issue in this study, though the sample was representative of the local population. However, lack of routine data collection at such services around migrant status meant that it was not possible to calculate the number of migrants who did not consent to participate in this research, nor to ascertain the number of migrants presenting at this service during the study period. We did, however, successfully engage a broad range of migrants (43 different nationalities) to participate in this feasibility study, which well represents the diversity of migrants we currently see in London.

Logistic challenges may exist in the implementation of routine multi-disease testing in this setting, for example in relation to the pathway between testing and delivery of samples to relevant laboratories for testing, although these would be relatively easy to overcome once routine screening was established. Screening is also challenging, since recording migrant status is not routine in this setting, which may mean eligible patients are missed, suggesting systems may need to be implemented to sensitively request patient information on migrant status to target screening. Implementation of screening will need to be tailored to each country context, given variations in emergency department infrastructure and facilities across Europe. There is a need for further research to examine the cost-effectiveness of screening in emergency departments.

## 5 Conclusions

This innovative multi-disease screening model was feasible to do in an emergency department context and facilitated the detection and treatment of LTBI, but highlighted the complexity of delivering LTBI screening to migrant populations, with greater emphasis needed on linkage-to-care and ensuring successful treatment outcomes. We identified major gaps in current screening provision to new migrants for a diverse range of key infections in the UK, which is a key consideration for all high migrant-receiving EU/EEA countries. Most recruited migrants had never been previously screened for an infectious disease since migration, despite previous engagement with primary-care providers. The emergency setting appears to be a feasible site for opportunistic multi-disease screening, which merits being tested further in larger-scale studies in the emergency room, as well as in other settings such as primary care. These studies could also consider screening for other infections highlighted in current guidelines [16], and include catch-up vaccination. New approaches to provision of preventive health care – including screening and vaccination – are needed to improve the health of migrants and their wider communities, and to ensure EU/EEA countries meet regional and global targets for the control and elimination of key infectious diseases.

## Contributions

SH and JSF designed and initiated the study and acquired the funding, with input from AK and AR in study set up at the service. LBN, CJ, JG did the intervention and LBN analysed the data. PP coordinated the analysis of samples. LBN and SH wrote the original draft, with input from all the authors.

## Data statements

All our data are freely available.

## Funding statement

This work was funded by the UK National Institute for Health Research Biomedical Research Centre (BRC) at Imperial College London, and the Imperial College Healthcare Charity. SH is funded by the NIHR (10.13039/100006662NIHR Advanced Fellowship NIHR300072) and the 10.13039/501100000691Academy of Medical Sciences (SBF005\1111). SH and LBN were supported by The 10.13039/100004440Wellcome Trust (209993/Z/17/Z), and the 10.13039/501100001704European Society for Clinical Microbiology and Infectious Diseases (ESCMID) through an ESCMID Study Group for Infections in Travellers and Migrants (ESGITM) research grant. LBN is funded by the 10.13039/501100000691Academy of Medical Sciences (SBF005\1047) and the MRC/AHRC/ESRC (MR/T046732/1). No funders had any role in the design of the study, in data collection, writing of the paper, nor decision to submit for publication.

## Ethical statement

All authors report no competing interests.

## Declaration of competing interest

All authors declare no conflicts of interest.
